# The survival rates and risk factors of implants in the early stage: a retrospective study

**DOI:** 10.1186/s12903-021-01651-8

**Published:** 2021-06-09

**Authors:** Yong Yang, Huiting Hu, Mianyan Zeng, Hongxing Chu, Zekun Gan, Jianmin Duan, Mingdeng Rong

**Affiliations:** 1 Department of Periodontology and Oral Implantology, Stomatological Hospital, Southern Medical University, Guangzhou, 510280 China; 2 Department of Stomatology, General Hospital of Southern Theater of People’s Liberation Army, Guangzhou, 510010 China

**Keywords:** Dental implant, Early survival rate, Risk factors, Multivariable logistic regression

## Abstract

**Background:**

Few large-sample studies in China have focused on the early survival of dental implants. The present study aimed to report the early survival rates of implants and determine the related influencing factors.

**Methods:**

All patients receiving dental implants at our institution between 2006 and 2017 were included. The endpoint of the study was early survival rates of implants, according to gender, age, maxilla/mandible, dental position, bone augmentation, bone augmentation category, immediate implant, submerged implant category, implant diameter, implant length, implant torque, and other related factors. Initially, SPSS22.0 was used for statistical analysis. The Chi-square test was used to screen all factors, and those with *p* < 0.05 were further introduced into a multiple logistic regression model to illustrate the risk factors for early survival rates of implants.

**Results:**

In this study, we included 1078 cases (601 males and 477 females) with 2053 implants. After implantation, 1974 implants were retained, and the early survival rate was 96.15%. Patients aged 30–60 years (OR  2.392), with Class I bone quality (OR  3.689), bone augmentation (OR  1.742), immediate implantation (OR  3.509), and implant length < 10 mm (OR  2.972), were said to possess risk factors conducive to early survival rates.

**Conclusions:**

The early survival rate of implants in our cohort exceeded 96%, with risk factors including age, tooth position, bone quality, implant length, bone augmentation surgery, and immediate implantation. When the above factors coexist, implant placement should be treated carefully.

## Background

As the living standard of the population improves, dental restoration has become the definitive therapy for most dental defects. Implants have been recognized as the ‘third set of teeth’, since they are beautiful, comfortable, and have good chewing efficiency, making them feel like natural teeth. Large-scale studies have reported that the long-term survival rates of implants are between 93.3 and 98% [1–3], indicating that dental implants are an effective treatment for edentulousness.

However, the failure of dental implants cannot be ignored. Manor and co-workers divided implant failure into early failure and late failure. Early failures are those that occur before the loading of masticatory forces, while late failures are those that occur after implants are loaded [4]. However, there is no specific definition for the timing of early implant failure. Most clinical studies have shown the early survival time of implants to be mainly concentrated in the first year or two after implantation, and most implant failures occur during early osseointegration and early mastication [2, 5, 6]. At present, there are few reports on the early survival of Chinese implants, and the reasons for failure have not been analyzed or summarized in detail. However, in recent years, the number of Chinese patients receiving implants has increased significantly. It is particularly important to study and analyze the risk factors for early implant failure in Chinese patients if such failure is to be effectively reduced and prevented. Therefore, we have studied and analyzed the many factors affecting both early failure and early survival rates of Chinese implants. At present, clinical studies at home and abroad report the possible risk factors as follows: patients’ general condition [7], local bone conditions in the implant area, patients’ bad habits [3], implant model, surgical placement technique [8, 9], early loading [10], and so on. Therefore, study and analysis of the main risk factors affecting the early survival rate of Chinese implants are crucial. Based on the preliminary analyses and statistics from our team, with a large sample size in the early stage, we found that the time of early implant failure was mostly concentrated in the first year after implant placement. Therefore, for better study and analysis of the risk factors of early failure in Chinese patients, we defined early survival as survival within one year after implantation [8, 9]. We retrospectively analyzed the early survival rates and the influencing factors of 2053 implants in our hospital from 2006 to 2017 to find the early survival rates of implants and determine the related influencing factors.

## Methods

### Study population

The present study incorporated all consecutive patients who underwent dental implant placement restoration over a period of one year at our institution between January 2006 and December 2017. The study protocol was approved by the institutional ethics committee (no. [2020] 92). Inclusion criteria were that: (1) all patients met the diagnostic criteria for a dentition defect; (2) the patients had no contraindications to surgery; (3) informed consent was provided; and (4) female participants were non-pregnant, non-lactating, and not menstruating. Exclusion criteria were that patients: (1) were receiving therapy in the head and neck region, had renal or liver disease, had uncontrolled diabetes mellitus, had chronic use of steroids, suffered from alcoholism, drug abuse, and local pathology or inflammation at the site of surgery, and had severe periodontal diseases; and (2) did not follow the doctor’s advice at the stage of osseointegration after implantation. Patients were told not to smoke after implant placement, especially during the wound-healing stage, so smoking was not included in the analysis. Patients who did not keep review appointments or respond to inquiries after placement were also excluded from the study. The end-point of the study was early survival rate, defined as the implant maintained in place and supporting the restoration within one year after placement [10, 11].

### Treatment

All implants placed in the dental practice were bone-level implants (rough, non-active, and acid-etched sandblasted surfaces), in which the Anthogyr connection is the inner triangle connection, the rest is the inner hexagon, and the Dentium and Anthogyr interface connections are designed by Cone Morse. Surgery was performed by three dentists who offered the complete range of dental treatment (called doctors A, B, and C). In China, they are all senior doctors with dental implant qualifications.

### Patient information collection and method

The surgeon completed the patient’s personal implant file immediately after the implantation procedure, recording the patient’s gender, age, implant position, bone quality, bone augmentation category, whether it was an immediate implant, whether it was submerged, implant category, diameter, length, implant torque, and general condition. According to the condition of bone in patients anticipating implant surgery, doctors chose the corresponding mode of operation: (1) stage I implant (abutment connection in the same period or stage II abutment connection); (2) stage I implant + bone augmentation; or (3) stage I bone graft + II implant. The superstructure was repaired by the implant surgeon from 3  to 6 months after implantation. All restorations were fixed, including single-crown, combined-crown, and fixed-bridge.

### Statistical analysis

Baseline characteristics were described. Categorical variables were presented as counts and percentages, according to gender, age, maxilla/mandible, dental position, bone augmentation, bone augmentation category, immediate implant, submerged implant category, implant diameter, length, implant torque, and other related factors. The Chi-square test was conducted to determine the differences between and among the groups. Variables associated with significant differences (p < 0.05) in Chi-square tests were subsequently introduced into the multivariate logistic regression model to further ascertain a simultaneous effect on failure rate. Analyses were performed with SPSS (Version 24) and GraphPad Prism 8. A two-sided significance level of 0.05 was used for hypothesis testing.

## Results

### Demographic information

1078 cases with 2053 implants were included in the study, with 601 males and 477 females. The average age of this cohort was 48.2 ± 0.6 years. After implantation, 1974 implants were retained, and the early survival rate was 96.15%.

### Time and cause of early implant failure

Early implant failure was defined as implant detachment or loosening within one year. Failure occurred in 37 implants within one month, in 32 implants within 2 to 4 months, and in 10 implants from 5 months to more than one year after restoration. Among the 79 unsuccessful implants, 69 failed before crown restoration and 10 failed thereafter. The main causes of early implant failure included infection (26 implants), excessive placement torque over 50 N/cm (11 implants), apical perforation involving perforation of the implant tip lip or buccal bone wall in second-stage surgery (9 implants), bone burn (8 implants), and improper occlusion due to early stress (8 implants). In addition, 4 implants failed due to periodontal disease of the adjacent tooth, 4 implants failed due to contact < 1 mm) too close to the root of the adjacent tooth, 3 implants failed due to poor oral hygiene habits or compliance, and 2 implants failed due to osteoporosis or insufficient boss mass. The remaining 4 failed for unknown reasons.

### Factors affecting the early survival rate

We investigated variables that might influence the early survival rate of dental implants. Patients of different genders had significantly different early survival rates, with a survival rate of 95.88% for males compared with 96.55% for females (Table 1). Dental position also influenced the survival rate. The survival rates of implants in anterior teeth, premolars, and molars were 93.33%, 95.28%, and 97.67%, respectively. As for bone quality classification, Class I enjoyed a survival rate of 90.90%, which was significantly lower than rates for Classes II and III (96.36%) and Class IV (97.41%). Different implant lengths had significantly different survival rates. The survival rates of implants shorter than 10 mm, between 10 mm and 13 mm, and longer than 13 mm were 90.91%, 97.13%, and 93.95%, respectively (Table 2). Implant strategies had a significant impact on early survival rates. Patients undergoing bone augmentation had implants with survival rates significantly lower than those in patients without bone augmentation (93.85% vs. 97.05%). Patients undergoing immediate implantation had significantly lower survival rates than those with delayed implantation (85.26% vs. 96.68%). Submerged implants had worse survival rates than others (94.28% vs. 97.40%) (Table 3). Other variables—including patient age, dentition, implant system, implant diameter, torque, and type of bone augmentation surgery—did not significantly influence survival rates.

**Table 1:** Patients’ characteristics that affected the early survival rate

	Survival (%)	Total implants (N)	*p* value
*Gender*			0.04*
Male	1163 (95.88)	1213	
Female	811 (96.55)	840	
*Age (years)*			0.443
< 30	241 (96.68)	241	
30–60	1305 (95.46)	1367	
≥ 60	436 (97.98)	445	
*Dentition*			0.272
Maxillary	882 (95.45)	924	
Mandibular	1092 (96.72)	1129	
*Dental position*			0.002**
Anterior teeth	322 (93.33)	345	
Premolar	439 (95.28)	461	
Molar	1213 (97.67)	1247	
*Bone quality*			0.018*
Class I	100 (90.90)	110	
Classes II–III	1723 (96.36)	1788	
Class IV	151 (97.41)	155	

*p < 0.05; **p < 0.01; ***p < 0.001

**Table 2:** Implant factors that affected the early survival rate

	Survival (%)	Total implants (N)	*p* value
*Implant system*			0.729
BEGO	666 (96.66)	689	
Anthogyr	841 (95.57)	880	
Osstem	64 (96.97)	66	
Dentium	350 (96.68)	362	
C-TECH	53 (94.64)	56	
*Implant length (mm)*			< 0.001***
< 10	240 (90.91)	261	
10–13	1554 (97.13)	1600	
≥ 13	180 (93.95)	192	
*Implant diameter (mm)*			0.33
< 3.75	436 (95.20)	458	
3.75–5.00	1425 (96.54)	1476	
≥ 5.00	113 (94.96)	119	
*Torque*			0.494
< 20	273 (95.12)	287	
20–40	1467 (96.51)	1520	
≥ 40	234 (95.12)	246	

*p < 0.05; **p < 0.01; ***p < 0.001

**Table 3:** Operation types that affected the early survival rate

	Survival (%)	Total implants (N)	*p* value
*Implant doctor*			0.05
A	898(95.03)	945	
B	504(97.11)	519	
C	572(97.11)	589	
*Bone augmentation*			< 0.001***
Yes	491 (93.85)	525	
No	1483 (97.05)	1528	
*Bone augmentation surgery*			0.51
Guided bone regeneration	190 (94.53)	201	
Bone compression	33 (91.67)	36	
Bone split	51 (98.08)	52	
Maxillary sinus floor lift	154 (92.22)	167	
Autogenous bone implant	63 (91.30)	69	
*Immediate implant*			< 0.001***
Yes	81 (85.26)	95	
No	1893 (96.68)	1958	
*Submerged*			< 0.001***
Yes	775 (94.28)	822	
No	1199 (97.40)	1231	

*p < 0.05; **p < 0.01; ***p < 0.001

### Multivariable logistic regression analysis

Variables that showed statistically significant differences in early survival rates were introduced into the multivariable logistic regression analysis, including patient gender, dental position, bone quality classification, implant length, the application of bone augmentation, whether implantation was immediate, and whether the implant was submerged. Implants not submerged and delayed implants showed protective factors for survival. Compared with patients aged older than 60 years, those aged 30 to 60 years had worse survival rates, while those younger than 30 years showed no statistically significant differences in survival rates. Compared with bone quality of Class IV implants, Class I was a risk factor for survival rate, while Classes II and III showed no significance. Other variables—including dental position (premolar, molar and anterior teeth) and whether bone augmentation was performed—did not show statistical significance relative to survival rates (Table 4).

**Table 4:** Multivariable logistic regression analysis for early survival rates

	OR	95 %CI	*p* value
*Age (years)*			
< 30	1.248	0.393–3.964	0.708
30–60	2.392	1.102–5.190	0.027*
≥ 60	Reference	Reference	
*Bone quality*			
Class I	3.689	1.029–13.224	0.045*
Class II–III	1.257	0.438–3.604	0.671
Class IV	Reference	Reference	
*Dental position*			
Anterior	Reference	Reference	
Premolar	0.981	0.467–2.063	0.960
Molar	0.544	0.258–1.145	0.109
*Implant length (mm)*			
< 10	2.972	1.211–7.296	0.017*
10–13	0.679	0.323–1.425	0.306
≥ 13	Reference	Reference	
*Submerged*			
Yes	Reference	Reference	
No	0.539	0.307–0.949	0.032*
*Immediate implant*			
Yes	Reference	Reference	
No	0.285	0.132–0.615	0.001***
*Bone augmentation*			
Yes	Reference	Reference	
No	0.990	0.555–1.764	0.972

*p < 0.05; **p < 0.01; ***p < 0.001

## Discussion

In China, a study of the early survival rates of clinical oral implants is of great significance, and such a study must of necessity be retrospective. However, an important factor in the study results is the rate of patient revisitation, which will gradually decrease as the observation period extends [11]. We chose the time point of one year after repair to conduct patient reviews, which greatly reduced the number of lost patients and increased the feasibility of this study. At the same time, to ensure the number of patients included in the study and reduce study error, we adopted methods of other clinical researchers [12]. Dental implants are a high-cost treatment in China, so when patients experience problems with the placement of their restorations, they will take the initiative to go to the hospital. Patients who did not keep return appointments were contacted by telephone and text messages. Our study reported the risk factors affecting early survival rates by detailed analysis of 2053 implants, and determined age (from 30 to 60 years old), bone quality (Class I), bone augmentation surgery, whether the implant was placed immediately or delayed, whether the implant was submerged during the first stage of surgery, implant length, and dental position as the risk factors for early survival. This is similar to the risk factors found by Antoun and co-workers [11]. According to the clinical literature reports and our research results, the above factors easily led to early implant failure for the following reasons:

(1) Chinese patients aged between 30 and 60 years old failed to maintain good oral hygiene due to the stress of work.

(2) Class I bone had a higher technical sensitivity to implant placement during site preparation of the implant socket, and high torque could cause bone loss during implantation, affecting the reconstruction of surrounding bone and causing buccal soft tissue retraction [6, 13].

(3) The presence of a bone augmentation surgery wound increased the likelihood of infection, resulting in the exposure and failure of implants. Visser et al. [14] also reported that implants with bone augmentation had a low retention rate and were less effective, especially when implanted at the same time as bone augmentation was performed [14–16].

(4) Because immediate implantation requires higher primary stability and sealing of soft and hard tissues and is more susceptible to bacteria and poor micromotion during healing, the risk of implant failure increased [17]. It has been reported that the risk of placement failure in an infected extraction socket was three times more than that of an infection-free extraction socket during immediate implantation [18]. Meijer et al. [19] reported a survival rate of 73.3% in implants placed immediately in the molar area. Ji et al. [20] found that delayed placement led to higher implant survival.

(5) In our submerged implants, the primary stability of the implants was insufficient, and the bone condition was poor, so bone augmentation was needed. Some studies have indicated that the survival rate of submerged implants was 97%, while that of non-submerged implants was 78% [21]. A systematic review showed that the failure risk of non-submerged implants increased by 2% within 6 months [22]. Of course, there are also literature reports that there was no difference in therapeutic effects between the two healing methods [23]. This may be caused by differences in the inclusion criteria and surgical methods in those studies.

(6) We found that short implants (less than 10 mm) failed easily, with low osseointegration efficacy and patients’ poor bone quality leading to the increased early failure rate. Chen et al. [24] found that the cumulative survival rate of short implants was 96.36%, which was slightly lower than the survival rate of standard implants (98.16%). Of the occurrences of short implant failure, 84.44% occurred in the early stage. The study by Krisam et al. [25] reported that the risk of early failure of implants shorter than 10 mm was 5.8 times more than that of longer implants (p = 0.0230), which was consistent with our results.

(7) The bone mass in the area of anterior teeth was often poor, and bone augmentation was often needed during implantation, leading to an increased risk of early implant failure in that location. The results of this study are consistent with those reported by Huang et al. [26].

In this study, the ability of the surgeon was not a high-risk factor. This is inconsistent with the results from Chrcanovic et al. [27], who reported that the characteristics of the individual surgeon had statistically significant effects on the cumulative survival of implants. It may be that these three senior implant doctors received systematic study and training before they performed implant procedures. Moreover, this study found that there was no statistically significant difference in the early survival rates of implants implanted by these three doctors. In addition, the group comparison according to the time of the implant specialty did not find that the implant survival rates of the doctors increased significantly with the extension of the implant time, but slightly decreased. There are several possible reasons for this result: With their continuing proficiency in and experience with implant technology, the three doctors will gradually expand the indications for surgery and attempt some cases with insufficient bone mass and high sensitivity to implant technology, thus increasing the risk of implant failure, resulting in a slight decline in the early survival rate of implants. Therefore, the key to reducing the rate of early implant failure is to strengthen learning and training, improving the diagnostic level of the doctors (the ability to evaluate implant difficulty and make a reasonable treatment plan) as well as their surgical implant techniques. This will guide us in preventing and reducing early implant failure in clinical practice and provide some referential experience for clinical implant counterparts.

Admittedly, this study had limitations. A detailed comparative study of the effects of systemic diseases and smoking on the early survival of implants has not been carried out, and some patients failed to report to the hospital for implant examination, which may have affected the rigor of the article. In future clinical research, we will design and implement the research plan more rigorously.

## Conclusions

The early survival rate of implants in our cohort exceeded 96%. The main reasons for failure were: the lack of osseointegration due to wound dehiscence and infection, bone burn, excessive torque placement, perforation of the fossa side wall during implant placement, early weight-loading, and uncontrolled periodontitis of adjacent teeth. Risk factors for early implant survival included age (patients from 30 to 60 years old), tooth position (anterior tooth area), bone quality (Class I), implant length (< 10 mm), bone augmentation surgery, and immediate implantation. When the above factors coexist, implant placement should be treated carefully.

## Data Availability

Most of the data generated or analyzed during this study are included in this published article. The datasets used and analyzed during the current study are available from the corresponding author on reasonable request.
